# Prognostic Effects of Adjuvant Chemotherapy-Induced Amenorrhea and Subsequent Resumption of Menstruation for Premenopausal Breast Cancer Patients

**DOI:** 10.1097/MD.0000000000003301

**Published:** 2016-04-08

**Authors:** Se Jeong Jeon, Jae Il Lee, Myung Jae Jeon, Maria Lee

**Affiliations:** From the Department of Obstetrics and Gynaecology, Seoul National University College of Medicine, Seoul, Korea.

## Abstract

Chemotherapy-induced amenorrhea (CIA) is a side effect that occurs in patients with breast cancer (BC) as a result of chemotherapy. These patients require special treatments to avoid infertility and menopause. However, the factors controlling CIA, resumption of menstruation (RM), and persistence of menstruation after chemotherapy are unknown. The long-term prognosis for premenopausal patients with BC and the prognostic factors associated with CIA and RM are subject to debate. We performed a retrospective study by reviewing the medical records of 249 patients with BC (stage I to stage III) who were treated with cytotoxic chemotherapy. The median patient age was 43 (range, 26–55 years) and the median duration of follow-up was 64 months (range, 28–100 months). The medical records indicated that 219 patients (88.0%) scored as positive for the hormone receptor (HR); the majority of these patients completed chemotherapy and then received additional therapy of tamoxifen. Our analyses revealed that 88.0% (n = 219) of patients experienced CIA, and the percentage of RM during follow-up was 48.6% (n = 121). A total of 30 patients (12.0%) did not experience CIA. Disease-free survival (DFS) was affected by several factors, including tumour size ≥2 cm, node positivity, HR negative status, and body mass index ≥23 kg/m^2^. Multivariate analysis indicated that tumour size ≥2 cm remained as a significant factor for DFS (hazard ratio = 3.3, *P* = 0.034). In summary, this study finds that the majority of premenopausal patients with BC (stage I to stage III) who receive chemotherapy experience CIA and subsequent RM. Although tumour size ≥2 cm is negatively associated with DFS, RM after CIA is not associated with poor prognosis.

## INTRODUCTION

Breast cancer (BC) is the leading cause of global cancer deaths among women. In 2008, 458,000 deaths were caused by BC, and there were an estimated 1.4 million new BC cases.^1^ Adjuvant chemotherapy and hormonal therapy for BC patients improves overall survival (OS) and disease-free survival (DFS). OS and DFS are improved for women younger than 50 years who receive ovarian ablation or suppression.^2^ Although ovarian ablation independently improves the outcome for patients with BC, it is not clear whether chemotherapy-induced amenorrhea (CIA) also improves the outcome for these patients. Walshe et al^3^ reviewed the CIA literature and reported that 41.7% of studies reported that CIA had a positive effect on survival, while 58.3% of the studies did not report a benefit. The International Breast Cancer Study Group recently published 2 clinical trials that evaluated the effects of chemotherapy on menstruation. These trials use chemotherapy to treat patients who had hormone receptor (HR) positive early BC and were premenopausal; the chemotherapy agents induce temporary or definitive ovarian suppression.^4,5^ The suppression of ovarian function plus either tamoxifen or exemestane (SOFT) trial used tamoxifen, whereas the triptorelin plus exemestane compared with triptorelin plus tamoxifen (TEXT) trial administered 2 chemical agents. The SOFT trial results were disappointing, in that the relative 17% improvement in DFS was not statistically significant. The TEXT trial showed that the combination with exemestane provided superior DFS at 5 years (22% improvement; *P* < 0.001). However, as observed in the SOFT trial, this did not translate into an OS advantage.

The CIA incidence in premenopausal patients with HR positive BC is directly related to patient age, and has been associated with type, schedule, and dosage of chemotherapeutic agent.^6–9^ Amenorrhea rates in the standard chemotherapy protocol used cyclophosphamide, methotrexate, and 5-fluorouracil (CMF regimen); for this treatment, the incidence of CIA ranged from 61% to 97% among older women (>40 years old) and from 18% to 61% among younger women.^3^ The NCIC CTG MA.5 trial used an anthracycline-based regimen and reported a higher CIA incidence than that of the CMF regimen.^10^ Regimens using paclitaxel and trastuzumab had lower CIA incidence (28% at 48 months) than those using standard cytotoxic agents.^11^

The Korean Cancer Registry indicates that women aged 45 to 49 years old have the highest incidence of BC. Of a total of 7359 new BC cases, 60% were diagnosed in premenopausal women and 21% were diagnosed in women younger than 40 years.^12^ Therefore, CIA incidence among BC patients is an important subject in Korea and in other countries with low fertility.^13,14^ CIA leads to problems such as infertility, osteoporosis, genitourinary dysfunctions, psychological distress, cardiovascular disease, and other acute conditions.^15^ However, CIA also reduces BC recurrence and improves survival, so it is not considered as a chemotherapy side effect. The addition of ovarian function suppression (OFS) to tamoxifen in the SOFT and TEXT trials caused adverse menopausal symptoms such as vaginal dryness, hot flashes, and reduced libido.^16^

Although CIA has been identified in BC survivors, it is unclear how resumption of menstruation (RM) occurs and whether it occurs in BC patients who receive chemotherapy. This study identifies factors associated with CIA incidence and assesses whether CIA and RM can be used as prognostic factors for premenopausal BC patients who receive surgery and adjuvant chemotherapy.

## PATIENTS AND METHODS

### Patient Selection and Clinical Characteristics

We performed a retrospective observational study using a cohort of patients who were referred for a regular gynaecological check-up after BC surgery at Seoul National University Hospital. The protocol used in this study was approved by the University Hospital Institutional Review Board. The eligibility criteria for inclusion in our study were as follows: patients presenting from January 2007 until February 2013; premenopausal BC patients who underwent surgical resection and subsequent adjuvant chemotherapy using anthracycline, taxane, or CMF; histological evidence for stage I to stage III BC; no previous chemotherapy, radiotherapy, or hormone therapy; and patients remain disease-free for the first 12 months after the start of chemotherapy (because CIA develops in a time-dependent manner). The following exclusion criteria were evaluated: other malignancies or bilateral BC; hysterectomy after chemotherapy; the adjuvant endocrine therapy included GnRH analogue treatment; a lack of information on menstrual history; or perimenopausal status (see below).

We defined CIA for premenopausal patients at the time of diagnosis as 6 consecutive months without a menstrual period according to Bines et al^17^ Transient CIA was defined as RM after CIA. Patient premenopausal status was defined as the last menstruation and regular menstrual cycles and the final menstruation within 3 months before the start of chemotherapy. RM was defined as regular cyclic bleeding that started more than 3 months after the start of CIA. Perimenopausal patients were defined as those whose last menstrual period occurred within twelve months before, but not within 6 weeks before, the start of chemotherapy. Perimenopausal patients were excluded from the study because spontaneous menopause could not be distinguished from CIA.

### Chemotherapy Regimens

The CMF regimen was administered to Patients who were diagnosed with 0 to 3 axillary lymph nodes received the CMF regimen by intravenous (i.v.) bolus injection on day 1 and day 8 of 500 mg/m^2^ cyclophosphamide, 50 mg/m^2^ methotrexate, and 500 mg/m^2^ 5-fluorouracil. Treatments were repeated every 4 weeks for a total of 6 cycles. Tamoxifen (20 mg daily) was given to patients with HR positive status for 5 years.

### Postsurgical Evaluation

After the completion of chemotherapy, patients were assessed every 6 months for 28 to 100 months (median = 64 months). The following 3 criteria were applied for our definition of DFS: the elapsed time from surgical resection to cancer recurrence, the elapsed time from surgical resection to the occurrence of a secondary primary cancer or death without evidence of recurrence, or the elapsed time from surgical resection to the last follow-up date. The recurrence of cancer was considered according to RECIST (version 1.1) criteria without histological confirmation as follows: typical liver or lung nodules as observed in imaging studies, or lytic bone areas as observed in radioisotope bone scans or radiographs.

### Statistical Methods

All experimental results were subjected to statistical analyses. Two-sided *P* values were computed with significance set at *P* = 0.05. Kaplan–Meier analyses were used to calculate DFS, and the log-rank test was used to compare DFS between groups. Multivariate analyses were performed for all prognostic variables using the time-dependent Cox's proportional hazard regression model. Potential predictive factors for CIA were evaluated using a chi-square test, 2-sided Fisher exact test, independent *t*-test, and logistic regression analysis. Statistical parameters were computed using SPSS v. 21.0 (SPSS Inc., Chicago, IL).

## RESULTS

The medical records of 258 patients who presented between January 2007 and February 2013 at Seoul National University Hospital with premenopausal BC were selected for this study. Cancer recurred within the first 12 months after chemotherapy for 9 patients, and these records were not included during further analyses. The median follow-up duration was 64 months (range, 28–100 months), and the median patient age was 44 years (range, 26–55 years). Patient baseline characteristics, tumours, and treatment regimens are summarised in Table 1. CIA occurred in 219 of 249 patients (88.0%). Amenorrhea often occurred during chemotherapy. The median time between the first amenorrheic episode (expected start date of period) and the date of starting the last menstruation before receiving chemotherapy was 4 months (range, 1–28 months).

**TABLE 1:**
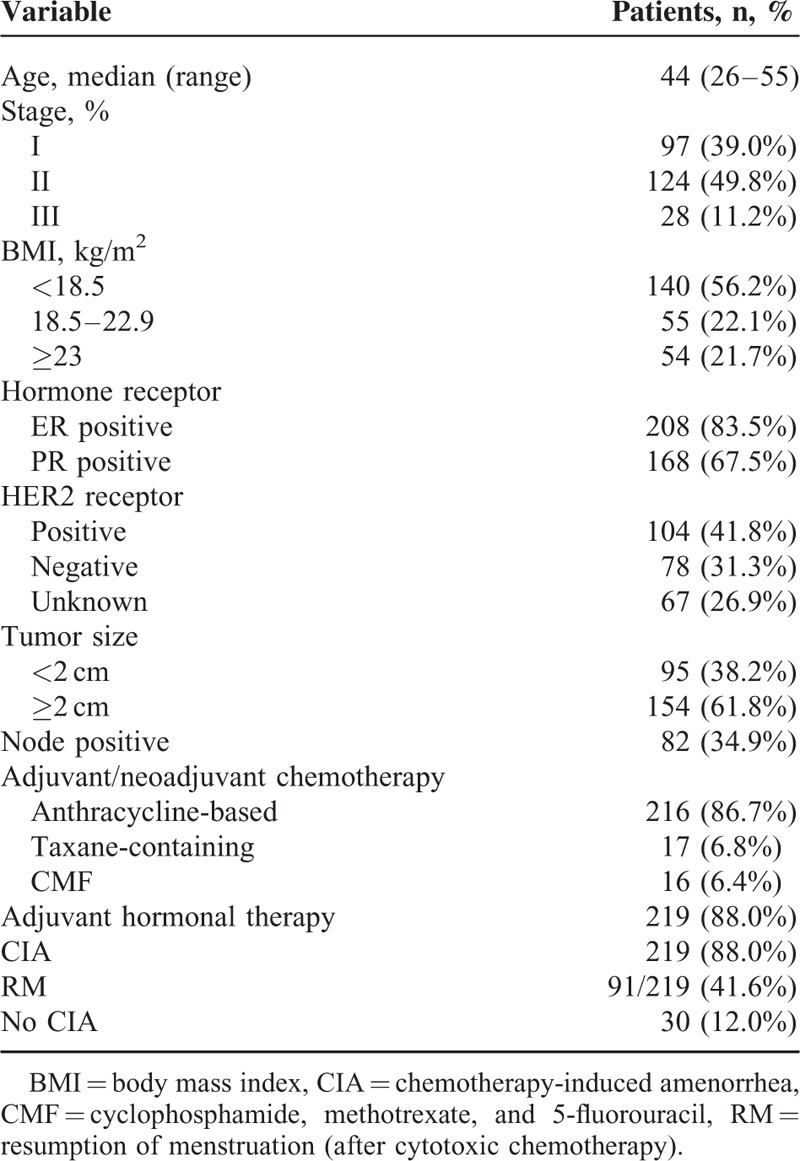
Clinical Characteristics of the Study Patients

The age-related incidence of CIA is presented in Table 2. Most (91.7%) CIA patients younger than 35 years experienced RM, while only 17.8% of CIA patients older than 46 years experienced RM. There were statistically significant associations between age and CIA (*P* < 0.001; Table 3) and between CMF regimen and CIA (*P* = 0.006). CIA was not associated with higher body mass index or tamoxifen therapy. Multivariate logistic regression analysis of predictive factors for CIA indicated that older age (>35 years) and CMF regimen were significantly associated with permanent CIA (Table 3).

**TABLE 2:**
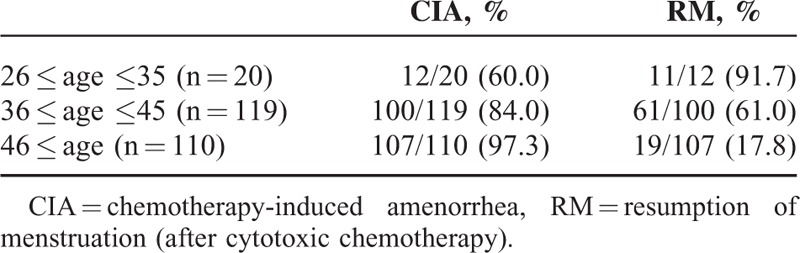
Incidence of CIA and RM According to Age

**TABLE 3:**
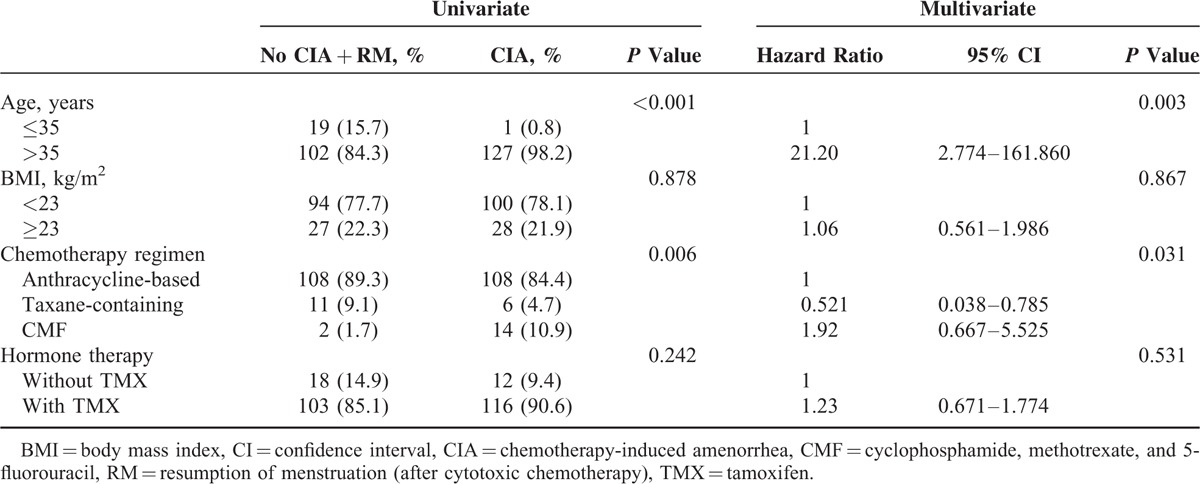
Univariate and Multivariate Analysis of Risk Factors for CIA

**FIGURE 1:**
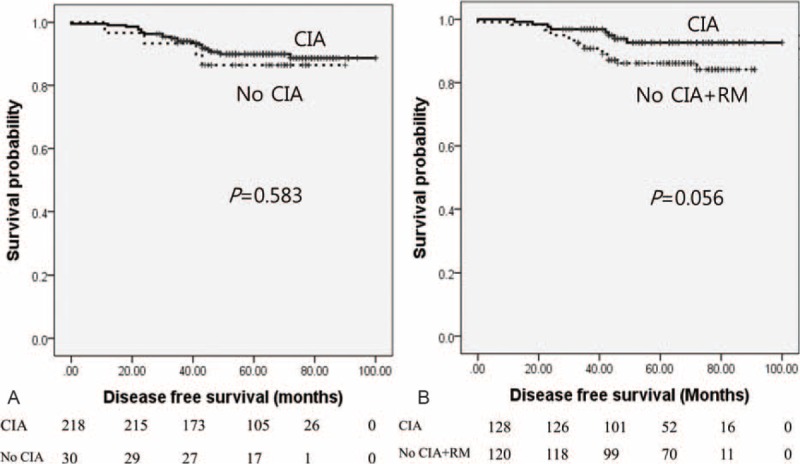
Kaplan–Meier analysis of disease-free survival for breast cancer patients experiencing CIA and RM. (A) CIA group versus group without CIA. (B) CIA group versus group without CIA + RM. All *P* values were calculated using the log-rank test. CIA = chemotherapy-induced amenorrhea, RM = resumption of menstruation.

We analysed correlations between DFS and clinical parameters (Table 4). For patients that did not experience CIA, DFS for 5 years was 85.0%, while DFS for patients who experienced CIA was 88.0%. HR negative patients with tumour size ≥2 cm, nodal involvement, and body mass index ≥23 kg/m^2^ had poor prognosis. Differences in DFS for patients with CIA versus patients with CIA and RM were not statistically significant (Figure 1A and B).

**TABLE 4:**
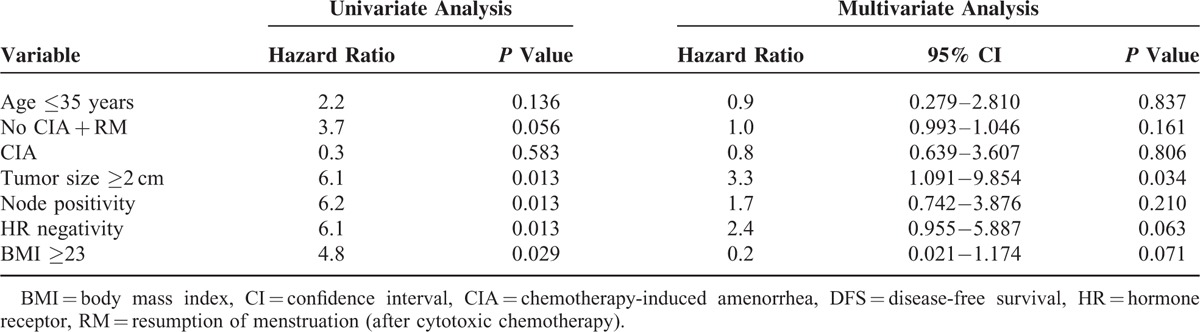
Univariate and Multivariate Analyses for DFS

A comparison of CIA, age, tumour size, nodal involvement, and HR status was performed using a Cox multivariate model. The results indicated that tumour size was significantly associated with poor DFS. RM showed a trend of shorter DFS, but this was not statistically significant.

**FIGURE 2:**
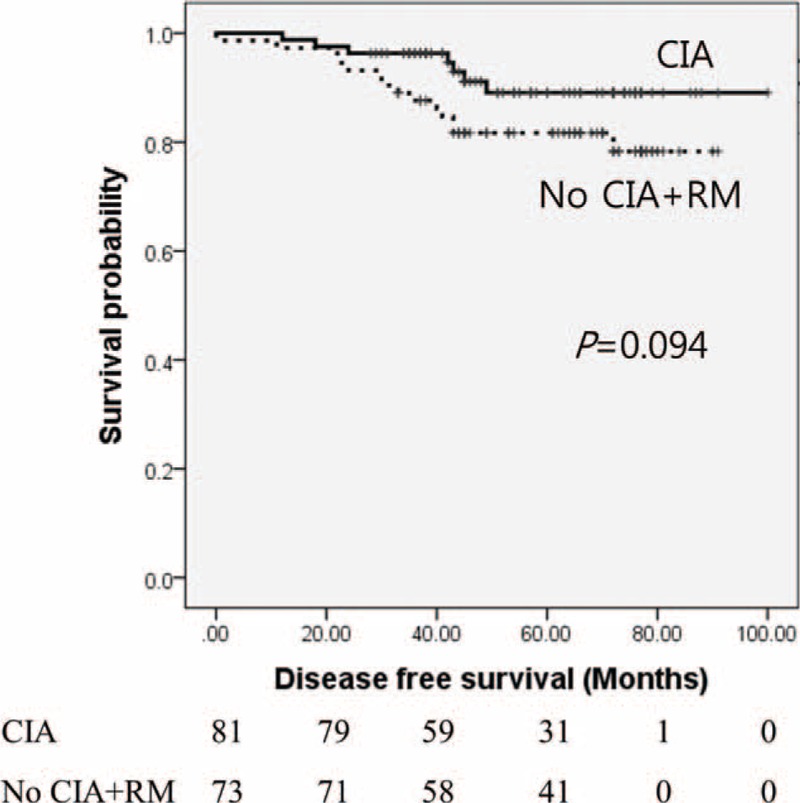
Kaplan–Meier analysis of disease-free survival for breast cancer patients experiencing CIA versus patients without CIA + RM and with tumour size ≥2 cm. Median survival was 67 and 90 months, respectively (*P* = 0.094). CIA = chemotherapy-induced amenorrhea, RM = resumption of menstruation.

Subgroup analyses were conducted for patients with tumour size ≥2 cm, and CIA was associated with longer DFS compared with that of no CIA and CIA with RM, although this difference was not statistically significant (hazard ratio 2.8, *P* = 0.094) (Figure 2).

## DISCUSSION

This study evaluated how the incidence and clinical significance of CIA affected the outcome of BC patients. The highest incidence of BC occurs in younger women in Korea compared with global averages, so a higher percentage of younger premenopausal patients receive chemotherapy after surgical resection for BC in Korea. For these younger patients, CIA is clinically important because of the associated decline in fertility.^12^ Multivariate analysis determined that predictive factors for CIA were older age and CMF chemotherapy regimen. Our study identified a strong correlation between CIA development and patient age. We found that the incidence of CIA was higher in older patients; CIA occurred in 70.4% of women younger than 40 years, and in 94.9% of women older than 40 years. These results are consistent with those of previous studies.^8–10,18,19^

There is some debate about whether tamoxifen affects CIA incidence. Some studies report that tamoxifen has no effect,^9,20^ while other studies report higher CIA incidence with tamoxifen.^18,21^ The NSABP-30 prospective clinical trial reported higher CIA incidence after 12 months of follow-up among women receiving chemotherapy with tamoxifen compared with that of chemotherapy alone (84% versus 60%, respectively).^18^ Our results were not consistent with those of NSABP-30, and temporary amenorrhea was observed primarily for patients receiving maintenance therapy with tamoxifen. The mechanism underlying this effect is not completely elucidated. Tamoxifen may increase the levels of circulating oestrogen, which may negatively regulate the hypothalamic-ovarian axis responsible for oestrogen synthesis. The CIA incidence may be affected by the regimen type.^6,22,23^ Some studies showed that higher cyclophosphamide doses correlated with higher incidence of CIA.^24,25^ A recent study reported that the amenorrhea incidence was 28% in premenopausal women treated with paclitaxel and trastuzumab.^11^ This shows that the CIA incidence is lower when the treatment regimens use chemotherapeutic agents with lower toxicity. In our study, CIA incidence was higher for patients treated with CMF chemotherapy, which is consistent with previously published results.

This study also evaluated the effects of CIA and RM on patient prognosis. The FUCHSIA Women's Study found that 41% of BC survivors experienced amenorrhea during treatment, but menses returned within 3 years after chemotherapy for 48% of survivors.^7^ In the current study, menses returned within 5 years after chemotherapy for 48.6% of survivors. Previous studies reported inconsistent results; however, some studies indicate that CIA may be an important prognostic marker for the outcome of HR positive BC patients.^3^ Our results indicate that longer DFS occurs for patients who experience CIA compared with those who do not experience CIA. However, this result was not statistically significant.

The SOFT investigators concluded that the addition of OFS with tamoxifen did not significantly improve DFS.^4^ The addition of OFS to tamoxifen did improve patient outcomes compared with those of tamoxifen alone for women with higher cancer recurrence risk who were treated with adjuvant chemotherapy and continued to be premenopausal. The TEXT study investigated which oral agent (tamoxifen or exemestane) was most effective in combination with OFS. The results indicated that exemestane improved DFS by 22% after 5 years, and there was a beneficial, although not statistically significant, trend in favour of the tamoxifen combination. In our study population, 88% of patients were HR positive; therefore, the beneficial effects conferred by CIA were relatively low. RM was moderately associated with shorter DFS. Consequently, RM effects might be more pronounced for patients considered as high-risk rather than low-risk. Chemotherapy effects would not be so pronounced for low-risk patients.

## CONCLUSIONS

This study identified CMF chemotherapy and age as the most important predictors of CIA and RM. DFS for BC patients appeared to be affected by RM. However, RM was not a factor for poor DFS prognosis for premenopausal BC (stage I to stage III) patients.
